# Prototype semantic infrastructure for automated small molecule classification and annotation in lipidomics

**DOI:** 10.1186/1471-2105-12-303

**Published:** 2011-07-26

**Authors:** Leonid L Chepelev, Alexandre Riazanov, Alexandre Kouznetsov, Hong Sang Low, Michel Dumontier, Christopher JO Baker

**Affiliations:** 1 Department of Biology, Carleton University, 1125 Colonel By Drive, Ottawa, Canada; 2 Department of Computer Science and Applied Statistics, University of New Brunswick, 100 Tucker Park Road, Saint John, Canada; 3 School of Computing, National University of Singapore, 21 Lower Kent Ridge Road, Singapore; 4 Institute of Biochemistry, Carleton University, 1125 Colonel By Drive, Ottawa, Canada; 5 School of Computer Science, Carleton University, 1125 Colonel By Drive, Ottawa, Canada

## Abstract

**Background:**

The development of high-throughput experimentation has led to astronomical growth in biologically relevant lipids and lipid derivatives identified, screened, and deposited in numerous online databases. Unfortunately, efforts to annotate, classify, and analyze these chemical entities have largely remained in the hands of human curators using manual or semi-automated protocols, leaving many novel entities unclassified. Since chemical function is often closely linked to structure, accurate structure-based classification and annotation of chemical entities is imperative to understanding their functionality.

**Results:**

As part of an exploratory study, we have investigated the utility of semantic web technologies in automated chemical classification and annotation of lipids. Our prototype framework consists of two components: an ontology and a set of federated web services that operate upon it. The formal lipid ontology we use here extends a part of the LiPrO ontology and draws on the lipid hierarchy in the LIPID MAPS database, as well as literature-derived knowledge. The federated semantic web services that operate upon this ontology are deployed within the Semantic Annotation, Discovery, and Integration (SADI) framework. Structure-based lipid classification is enacted by two core services. Firstly, a structural annotation service detects and enumerates relevant functional groups for a specified chemical structure. A second service reasons over lipid ontology class descriptions using the attributes obtained from the annotation service and identifies the appropriate lipid classification. We extend the utility of these core services by combining them with additional SADI services that retrieve associations between lipids and proteins and identify publications related to specified lipid types. We analyze the performance of SADI-enabled eicosanoid classification relative to the LIPID MAPS classification and reflect on the contribution of our integrative methodology in the context of high-throughput lipidomics.

**Conclusions:**

Our prototype framework is capable of accurate automated classification of lipids and facile integration of lipid class information with additional data obtained with SADI web services. The potential of programming-free integration of external web services through the SADI framework offers an opportunity for development of powerful novel applications in lipidomics. We conclude that semantic web technologies can provide an accurate and versatile means of classification and annotation of lipids.

## Background

Lipids and their metabolic derivatives play a crucial role in the biology of many living organisms. Imbalance or abnormality in lipid metabolism often accompanies diseases such as Alzheimer's disease, Mycobacterium infections, and cancer. In order to attain a better understanding of the role of lipids in physiological processes, scientists have turned to high throughput technologies and system-level approaches to analyze the lipid composition of living organisms, in a field broadly termed lipidomics [1]. Lipidomics generates a large amount of heterogeneous chemical and biochemical data that must be integrated and analyzed in a systematic manner. Such efforts are, however, hampered by the lack of consistent lipid classification.

Classification is a core activity in biomedical investigation [2-6] and the robustness of a classification scheme clearly has profound consequences on the interpretation of scientific experiments and data analyses. It is important to distinguish between annotation and classification within this study, since the lines between the two activities may effectively vanish in certain contexts. While the results of classification can be used to annotate chemical entities with class membership information, in this work we shall regard annotation as a distinct activity from classification because we shall use molecular annotation information that is not an outcome of classification in order to enable automated classification of molecular entities.

For lipids, as for many other chemical entities, classification is extremely important because it helps predict the biological activity of a given molecule based on that of related molecular classes, since biological functionality is a direct consequence of molecular constitutional and structural configuration. This knowledge can be used when searching for drug targets, bio-transformation candidates, or in constructing metabolic and retro-synthetic pathways [7]. In current practice, lipids can be systematically classified according to various features, such as atomic connectivity, physicochemical properties, characteristic functional group presence, biological activity, biosynthetic pathway, or organism/system of origin. Few of these classification schemes have explicitly stated consensus criteria or rigorous definitions for membership of specific lipid classes, let alone machine-understandable ones. This further exasperates the existing challenges in the understanding and sharing of lipid-related knowledge. Consequently, lipid nomenclature has yet to become a robust research tool for lipidomics and lipid-related fields.

The de facto standard central repository of lipid molecule data, LIPID MAPS [6], primarily uses is-a relationships to categorize lipids, with many definitions describing LIPID MAPS lipid classes remaining implicit. Moreover, when classes within this database (and other hierarchy-oriented databases, such as ChEBI [2]) are defined, it is often done with a diagram in the form a molecular graphic that requires a trained lipid expert to be able to interpret and use it for manual classification tasks. No definition, independent of the diagram and corresponding chemical structure, exists and the diagram is not flexible, or extensible. A rigorous definition demands a declaration of minimal necessary and sufficient conditions for class membership that could adequately describe a lipid without referring to a molecular structure diagram. To constructively address the absence of a formal definition, lipids must be defined in a manner that is systematic, yet at the same time explicit and machine-interpretable. While numerous languages are available to represent knowledge, the emergence of the Semantic Web as a collection of standards that extend current web technology is finally allowing us to represent, publish, share, reuse, query, interconnect, and reason about both data and expert knowledge. Of particular interest is the Web Ontology Language (OWL) which provides an expressive and decidable subset of first order logic that enables the construction of formal ontologies for executing automated reasoning tasks that require inferences to check data consistency, undertake classification and answer queries. Recent progress in the development of formal ontologies for lipids in OWL [8,9] has made it possible to define lipids in a manner that is systematic, yet at the same time semantically explicit, enabling machine reasoning about lipids.

### Semantic Web Technologies for Lipidomics

The Lipid Ontology, LiPrO [8], captures knowledge about lipid types and their structural features. In LiPrO distinct combinations of functional groups underpin the axiomatic definitions of lipid types, and they are asserted as both necessary and sufficient (i.e. class equivalence) in order to support automated classification. The Lipid Ontology contains approximately 400 lipid definitions that rely on the precise combinations of functional groups, based on the mereological relationships (parthood) between a compound and its functional groups [10]. For example, in LIPID MAPS, hydroxy/hydroperoxyeicosatrienoic acids are defined as molecules containing exactly three alkenyl functional groups, as well as a hydroxy or a peroxy functional group, a carboxylic acid group, and a primary alkyl chain. By formalizing these class restrictions as an equivalent class axiom, it becomes possible to use automated reasoning to infer class membership for an instance of a lipid, based on functional group annotation data.

However, a framework consisting solely of functional group annotation and chemical classification alone, our primary use case, does not adequately address the full spectrum of problems in lipidomics. For instance, a lipid researcher may not only be interested in the class of a given lipid, but also its biochemical role, putative interacting proteins, and various chemical metrics useful for rationalizing the biochemical activity of the lipid. The ability to integrate, analyze, and synthesize disparate information on the fly is especially desirable in investigations where rapid screening of thousands of novel compounds is necessary to identify entities of potential commercial or practical significance.

The problem of integration of disparate data in cheminformatics and bioinformatics has, to some extent, been addressed by the encoding of chemical and biological data using the Resource Description Framework (RDF) [11], a base-level formal language for the Semantic Web. In RDF, knowledge is captured in the form of collections of statements represented as subject-predicate-object triples. Repositories of triples, or triple stores, contain explicit relationships between the various biochemical entities (e.g. binding partners, small molecule roles in reactions), as well as information about their measured and computed characteristics. Using the SPARQL query language, triple stores can be queried for entities that have a particular combination of attributes and relationships. Taken together with a formally defined ontology of types and relationships, new facts can be inferred using automatic reasoning. Already, several early applications have successfully demonstrated the power and the versatility of this approach to knowledge integration in the life sciences as well as assisting drug discovery [12,13].

In the vast majority of cases, however, relational databases remain the predominant storage medium for life sciences data and difficulties in answering a particular query from such biochemical knowledgebases may sometimes arise due to missing data or insufficient concept mapping. Such data and relations could potentially be derived automatically, but the poor connectivity and interoperability of the various software packages required for such data repopulation frequently necessitates the involvement of human experts. Another manifestation of semantic web technologies provides a solution for problems of software interoperability, specifically for integration of biomedical resources, using semantic web services. The Semantic Annotation, Discovery, and Integration (SADI) framework [14] makes it possible to automatically integrate database queries with semantic web service-enveloped computational components and automatically construct elaborate computational workflows through a single SPARQL query, with no additional programming or manual software matchmaking on the part of the researcher.

### Semantic Automated Discovery and Integration Framework

SADI is a framework for creating Semantic Web Services. It comprises of a set of conventions that make it possible to create HTTP-based services that are semantically described and that can be automatically discovered and orchestrated. Two key features are that i) SADI services work by providing additional information about the input data they consume and ii) they use RDF documents as the medium of exchange. Service inputs and outputs are described semantically as classes described using the Web Ontology Language (OWL). The service responds to an input RDF document if the subject can be inferred to be an instance of the service's specified input class. The service is then expected to generate new information, as specified by the output class, which it associates to the subject. Thus, the output class specifies what new annotations may be added by the service.

An important part of the SADI framework is a freely accessible and expandable registry in which services and their descriptions are indexed and through which services are exposed for discovery and automated composition. Because the input class, the output class, and the predicates corresponding to the annotation placed upon the subject in the input are known and formally defined, it is possible for an external client to automatically reason over the available services and identify services that have to be called in order to complete a given query. For example, consider a service that receives as input a protein identifier (e.g. UniProt identifier) and produces as output its corresponding protein sequence. The inputs of the SADI service will correspond to a class 'protein' in an external ontology (that contains concepts used in service description) which has a 'UniProt identifier' as an attribute. The output of the SADI service will have 'amino acid sequence' as an attribute.

Formal semantic service descriptions in SADI facilitate completely automatic discovery and composition of SADI-compliant web services by clients with machine reasoning capability. SHARE [15,16] is one such experimental client designed to perform automated discovery and service orchestration. SHARE receives SPARQL queries as input and executes them by first discovering and then invoking suitable SADI services from an online SADI registry. For example, a query to compute the molecular weight of a protein given its UniProt identifier may involve the retrieval of the corresponding amino acid sequence based on the identifier from UniProt, followed by the calculation of the molecular weight of this sequence. If available, services and/or data will be used to form an uninterrupted chain linking the instance of the input class to the desired output class or annotation requested in the SPARQL query.

A typical SHARE user must first determine the entity (or entities) they are starting with or asking about, and then determine what information they would like to obtain. The user then composes a SPARQL query using the types and relations present in the SADI registry. The SHARE engine decomposes the query and identifies the set of services that are relevant to the query, and then generates and executes a query plan. Thus, the user needs only to formulate a coherent query. In recent work we leveraged the interoperability and facile integration of SADI services using the Web-based SHARE client in a bioinformatics use case in the domain of Mutation Impact Mining [17,18].

In this work, we explore the construction of a prototype lipid classification and annotation pipeline to support lipidomics research by integrating small molecule structural and physical annotation, classification, and knowledge mining services within the SADI framework. We describe the automated classification through (i) the invocation of a service which is capable of annotating and enumerating the functional groups present in a given input molecule through substructure searching on the molecular chemical graph structure, as represented by the corresponding SMILES string [19], and (ii) a classifier service that assigns chemical entities to appropriate ontology classes by reasoning over class description in the LiPrO ontology and checking them against the set of chemical subgroups provided by the structure annotation service. Overall, our framework takes as its input an RDF-encoded description of the chemical graph of an arbitrary lipid and provides as output RDF functional group annotation information on this lipid, as well as its class membership information, also encoded in RDF. The utility of our functional group annotation and lipid classification services lies in the ability to provide a semantic description for newly discovered lipids for which no class is currently assigned. Moreover, these services also provide the opportunity to review existing classifications based on expert manual curation, such as that of the LIPID MAPS, which in our automated system are based exclusively on structural attributes.

## Results

### Overview

**Figure 1:**
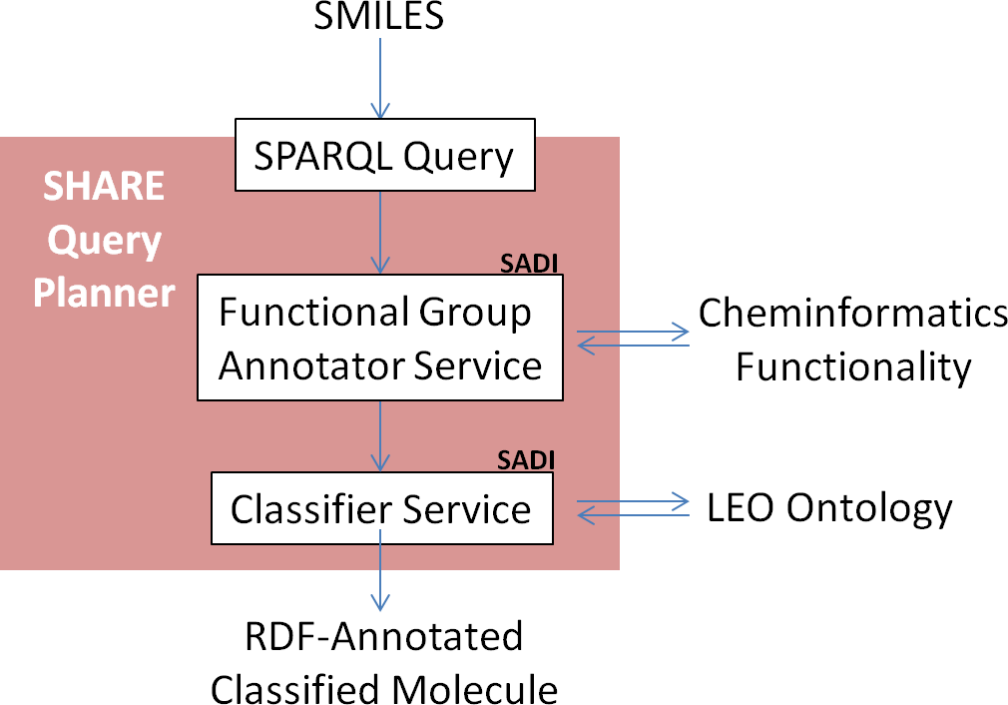
**Summary of the proposed core lipid classification framework**. A SMILES description of molecular graph for a given chemical entity is submitted to the SHARE client, which automatically identifies and orchestrates the execution of the annotation and classification SADI services. The output is an RDF graph that contains classification and functional group annotation of the input chemical entity.

Our work is motivated by the need for integrated query access to distributed services that compute information about lipids. Here we describe services that, starting with a SMILES representation of the lipid molecule, provide functional group annotations of molecules, and classify molecules according to these annotations (Figure 1). We illustrate, in conjunction with auxiliary SADI services, the integration of inferred class information with (i) information relating to proteins that interact with lipids belonging to the inferred lipid types and (ii) literature references relevant to the class of the lipid under investigation. To assess the quality of our framework, we document the performance of our classification service on a small subset of lipids, namely of eicosanoids, found in the LIPID MAPS database. Finally, for each analyzed LIPID MAPS eicosanoid molecule, we contrast the semantic definition of lipid classes in our ontology, with the class description in the LIPID MAPS database.

### Integration of Lipid Resources with SADI and SHARE

The first and the most important step in constructing our prototype pipeline is the development and integration of SADI-based annotation and classification semantic web services. The functional group annotation service [20] consumes a molecule whose structure is specified as a SMILES string (Listing 1) and annotates it with the set of unique occurrences of functional groups found in the molecule through subgraph isomorphism detection (Listing 2). In the input, a molecule is related to its SMILES descriptor through the 'has attribute' relation (SIO_000008) as defined by the Semantic Science Integrated Ontology (SIO) [21]. Here and elsewhere in this work, the sio prefix shall refer to this ontology (see Methods). The descriptor itself is an instance of the type CHEMINF_000018 (also found in SIO) and is linked to its string value through the 'has value' datatype (SIO_000300). In the output of the functional group annotation service, a molecule is related to a given functional group through the 'has proper part' predicate (SIO_000053).

**Listing 1**. A fragment of N3 RDF input to the functional group annotation service.

@prefix ss: <http://semanticscience.org/>.

@prefix sio: <http://semanticscience.org/resource/>.

ss:LMFA03010001 sios:SIO_000008 ss:LMFA03010001smiles.

ss:LMFA03010001smiles

rdf:type sio:CHEMINF_000018;

sio:SIO_000300 "CCCCC[C@H](O)/C=C/[C@H]1[C@H](O)C[C@H](O)[C@@H]1CC(=O)CCCCC(=O)O".

**Listing 2**. A fragment of N3 RDF output of the functional group annotation service.

<http://semanticscience.org/LMFA03010001>

sio:SIO_000053 [a lipro:Propyl].

sio:SIO_000053 [a lipro:Alcohol].

. . . . .

sio:SIO_000053 [a lipro:Hydroxy_Compound].

In turn, the lipid classification service [22] consumes a molecule annotated with functional groups and produces annotation in the form of its LiPrO classification through the predicate lipidHasLiProClass which is a subproperty of rdf:type and is defined in the supporting service ontology [23], abbreviated as ont:

<http://semanticscience.org/LMFA03010001> ont:lipidHasLiProClass lipro:LC_Prostaglandin.

While one could write a script to sequentially call the two services, this can also be automatically carried out by the SHARE client with an appropriate SPARQL query (Listing 3). Please note that in the interest of clear presentation, the queries presented here are truncated. The full, functioning queries, along with detailed instructions for executing them, are available [24].

**Listing 3**. A fragment of SPARQL query for classification of a lipid using the SHARE client. Full query is available [24].

[L1] SELECT DISTINCT ?liProClass ?LMClass ?LMClassId

[L2] FROM <http://unbsj.biordf.net/lipids/service-data/LMFA03010001.rdf>

[L3] WHERE

[L4]   {

[L5]      <http://semanticscience.org/LMFA03010001> lco:lipidHasLiProClass ?liProClass.

[L6]      <http://semanticscience.org/LMFA03010001> lmo:lipidHasLMClass ?LMClass.

[L7]      # 'has attribute'

[L8]      ?LMClass sio:SIO_000008 ?LMClassIdRes.

[L9]      # 'identifier'

[L10]      ?LMClassIdRes rdf:type sio:SIO_000115.

[L11]      # 'has value'

[L12]      ?LMClassIdRes sio:SIO_000300 ?LMClassId

[L13]   }

To execute the query, SHARE first obtains the SMILES information from the RDF file identified in the "FROM" clause (whose contents are shown in Listing 1). It then calls the annotation service with the SMILES string found in the input and obtains the functional group annotation. Having detected the compatibility of the annotation service output and the classification service input type, the client then calls the classifier service by providing the functional group annotations and obtains the identifiers of the LiPrO classes that match the given lipid. In addition, it discovers the LIPID MAPS classes that would apply to the lipid being classified (lines [L6]-[L12]). This is achieved by calling a third service [25] that simply consumes the identified LiPrO types and maps them to the corresponding LIPID MAPS types. There is absolutely no programming involved, nor any computer-aided workflow composition: the query is declarative and can be created by a user with no programming experience.

This query returned the molecule classified into two LiPrO classes, LC_Prostaglandin and LC_Isoprostane. The definitions of these classes match the structure of the original molecule in terms of participating functional groups and conform to the informal definitions of the corresponding LIPID MAPS types FA0301 and FA0311. Although rigorous query performance evaluation is not among the goals of our exploratory study, we would like to mention, for illustrative purposes, that the execution of the query as indicated took less than one minute. Most of this time was spend by the SHARE client automatically constructing the workflow, whereas each of the service calls took less than one second.

### Integration of Lipid Class Information with Reference Literature

Given lipid class information, it is now possible to identify the biological significance of the lipid under investigation by automatically mining the relevant literature for the roles of lipids belonging to this class. In a related effort, we have been exploring the use of text mining for the extraction of information from scientific literature as it pertains to lipids and related biological entities, such as proteins. We created a SADI service to carry out text mining (see Methods), making it possible to identify lipids mentioned in text corpora composed of the latest scientific literature. This service consumes the document location (specified as a URL) and annotates it with corresponding LiPrO lipid classes using SIO's 'is referred to by' relation that links entities to sources of information about them. To support our experiments, text mining has been performed for a small set of published work in lipid-related research and stored in an RDF triple store, which will be later extended to accommodate a more comprehensive body of literature. An additional service, the Lipid Literature DB service, provides the LiPrO annotation for a given publication by retrieving the text mining results from this triple store [26]. This service accepts a LiPrO lipid class as input and can identify the instances of this class and its subclasses within published documents. By combining all these services, it becomes possible to not only classify a given lipid, but also find information about its potential biological activity within published literature. For example, starting from a simple SMILES description of an eicosanoid, we can identify it as eicosapentaenoic acid, an essential fatty acid, and find the relevant literature (Listing 4).

**Listing 4**. A truncated literature retrieval query. Full query is available [24].

[L1] SELECT DISTINCT ?liProClass ?document ?LMClass ?LMClassId

[L2] FROM <http://unbsj.biordf.net/lipids/service-data/LMFA03010001.rdf>

[L3] WHERE

[L4]   {

[L5]      <http://semanticscience.org/LMFA03010001> lco:lipidHasLiProClass ?liProClass.

[L6]      <http://semanticscience.org/LMFA03010001> lmo:lipidHasLMClass ?LMClass.

[L7]      #      'has attribute'

[L8]      ?LMClass sio:SIO_000008 ?LMClassIdRes.

[L9]      #            'identifier'

[L10]      ?LMClassIdRes rdf:type sio:SIO_000115.

[L11]      #         'has value'

[L12]      ?LMClassIdRes sio:SIO_000300 ?LMClassId

[L13]      #         'is referred to by'

[L14]      ?liProClass sio:SIO_000212 ?document.

[L15]   }

### Identification of Related Proteins Based on Lipid Type

Our final use case considers linking the lipid under investigation to potentially related proteins in metabolic or signalling pathways. This is achieved by formulating a SHARE client query that automatically discovers, invokes, and integrates the output of five very different SADI-compliant services. For protein interaction information, we rely on the LIPID MAPS Proteome DB (LMPD) [27], which links lipids to proteins that are involved in lipid metabolism or interactions.

We created a service that simply uses LMPD to provide UniProt entries for proteins related to a specified lipid category [28]. This service links a lipid within an identified high-level LIPID MAPS category (e.g. fatty acids) to its protein interaction partner through the SIO's 'is related to' relation. However, this service is not strictly semantically interoperable with our classifier service. While the classifier service produces the closest matching LiPrO lipid class and another service maps LiPrO type annotations to LIPID MAPS type annotations [24], the higher-level lipid classes required by the protein retrieval service are not explicitly stated. To address this, we have created another LIPID MAPS-based service to take a URI denoting an arbitrary class in the LIPID MAPS nomenclature, for example, a lower-level class like Prostaglandins (FA0301), and to compute the LIPID MAPS higher-level category that contains this class, which for Prostaglandins is Fatty Acyls (FA) [29]. A simple SPARQL query prompts SHARE to integrate this higher-level class information with inferred lipid classification information (Listing 7).

**Listing 7**. A truncated SPARQL query to identify proteins related to the lipid under investigation. Full queries are available [24].

[L1] SELECT DISTINCT ?liProClass ?LMClass ?LMClassId ?LMCategory ?UniProtRecord

[L2] FROM <http://unbsj.biordf.net/lipids/service-data/LMFA03010001.rdf>

[L3] WHERE

[L4]   {

[L5]      <http://semanticscience.org/LMFA03010001> lco:lipidHasLiProClass ?liProClass.

[L6]      <http://semanticscience.org/LMFA03010001> lmo:lipidHasLMClass ?LMClass.

[L7]      #      'has attribute'

[L8]      ?LMClass sio:SIO_000008 ?LMClassIdRes.

[L9]      #         'identifier'

[L10]      ?LMClassIdRes rdf:type sio:SIO_000115.

[L11]    #    'has value'

[L12]      ?LMClassIdRes sio:SIO_000300 ?LMClassId

[L13]      ?LMClass rdfs:subClassOf ?LMCategory.

[L14]      #      'is related to'

[L15]      ?LMCategory sio:SIO_000001 ?protein.

[L16]      #      'is about'

[L19]      ?UniProtRecord sio:SIO_000332 ?protein }

Here, lines up to [L12] are our base case: we essentially obtain a LIPID MAPS low-level class URI in the variable LMClass. Line [L13] maps this class to the corresponding higher-level LIPID MAPS category and line [L15] subsequently retrieves related instances of related proteins, which is done by calling the corresponding service [28]. The auxiliary constraint in line [L19] extracts the URL of the corresponding UniProt record from the protein description returned by this service. A test run with SHARE terminates in less than a minute and returns a list of 603 proteins corresponding to fatty acyls (FA), which is the common LIPID MAPS category for prostaglandins and isoprostanes to which the lipid is classified. For this input, we have received, among others, the URLs for the UniProt records P97363, a palmitoyltransferase, and P55249, a lipooxygenase, both of which are known to be involved in fatty acid metabolism. Thus, we have been able to integrate five different services to take our prototype pipeline from a simple SMILES specification of a lipid to its biological roles found in literature and the potential protein partners.

### Evaluation of Lipid Classification Performance

The accuracy of lipid classification that we are capable of achieving defines to a great extent the applicability of our entire prototype lipidomics infrastructure. Within a randomly selected but representative sample of 150 eicosanoid lipids, our combination of annotation and classification services assigned a class consistent to the LIPID MAPS asserted classes in 96% of lipid entities considered. This figure includes about 8% of all lipids that received classification to a more general class in the classification hierarchy than the target class, while still being a correct classification. Furthermore, approximately 4% of lipids received a classification that was entirely incorrect, and 7% of all lipids were classified into a LIPID MAPS-consistent and LIPID MAPS-inconsistent class simultaneously. For instance, in case of the LIPID MAPS record LMFA03010001, that was correctly classified as a lipro:LC_Prostaglandin and the corresponding LIPID MAPS class FA0301, this lipid was also classified as a lipro:LC_Isoprostane (LIPID MAPS class FA0311), which is incorrect. This is because additional non-structural information is required, namely synthetic route, to differentiate the two classes.

Misclassification was not always a reflection of insufficient class definitions in the supporting ontology. Despite manual curation of the LIPID MAPS database, it appears that the database is not currently entirely error-free. For instance, compounds LMFA03050020 and LMFA03050021 were classified as epoxyeicosatrienoic acids by our automated classification framework and as hydroxy/hydroperoxyeicosatrienoic acids in the LIPID MAPS database. In the Lipid Eicosanoid Ontology, hydroxyl/hydroperoxyeicosanoic class (LIPID MAPS FA0305) is specified using the following equivalent class expression.

'has proper part' exactly 1 'primary acyl chain'

and 'has proper part' some 'carboxylic acid'

and 'has proper part' some (Alcohol or Hydroperoxide or Peroxide)

and 'has proper part' exactly 3 'Alkenyl_Group'

Similarly, the class of epoxyeicosatrienoic acids, equivalent to LIPID MAPS class of the same name (FA0308) is specified in LEO with the following expression.

'has proper part' exactly 1 Primary_Acyl_Chain

and 'has proper part' some Epoxy

and 'has proper part' some Carboxylic_Acid

and 'has proper part' exactly 3 Alkenyl_Group

Using our functional group annotation service, both of these acids were found to contain (among other non-diagnostic groups) a combination of three alkenyl functional groups, a carboxylic acid functional group, and one epoxide on the main chain, without the alcohol, hydroperoxide, or peroxide functional groups necessary to classify them into FA0305. This suggests that these two fatty acids should, in fact, be reassigned into a different class in the LIPID MAPS database, namely into the epoxyeicosatrienoic acid class, FA0308. Thus, explicit class definitions in our lipid ontology have allowed us to efficiently and automatically identify and correct an error in the expert-curated database.

## Discussion

Fuelled by massive amounts of data derived from disparate experimental procedures, research in biology and chemistry has become increasingly interdisciplinary. In contrast there are relatively few examples of consensus data and knowledge representation formats or frameworks that can truly integrate the computational, literature, and experimental resources necessary for success in interdisciplinary fields like systems science. The prototype framework presented in this paper has demonstrated the power of self-organizing semantic web services in addressing a highly interdisciplinary problem. In this work, we have been able to successfully integrate computational and informational resources from disparate disciplines to produce a single prototype framework for lipidomics research that requires no special skills from the end user, other than the ability to formulate standard SPARQL queries. In the process, we have been able to make the case for the use of OWL ontologies in the construction of consistent, reproducible, and flexible chemical hierarchies that leverage an advanced range of logical constructs from the OWL language.

### Integration with Broader Web Service Networks

The prototype pipeline we have described has significant potential for extension and further refinement, as additional services become available. For example, the current chemical classification service need not be constrained solely to structural measures since the integrative nature of SADI allows for the flexible definition of chemical classes based on multiple features of the classes in question and subsequent automated invocation of the appropriate set of SADI-compliant services. A library of SADI services computing many chemical metrics and properties is already available [30] and can be readily invoked to define a wide range of chemical classes [31] if these properties are stipulated in the formal class definitions in the lipid ontology. Furthermore, additional services can be integrated into this framework to address queries involving predicted lipid metabolism, retrieval and clustering with similar chemicals, or even rapid docking of lipids to the collection of the potentially interacting proteins that we are capable of identifying with this prototype pipeline. In short, the applications and extensions of this work are only bounded by the knowledge and the services that can be represented and implemented consistently within the context of the Semantic Web.

### Limitations of the Proposed Approach

The experiments with the lipid classification services presented here have confirmed some of the limitations of the SADI framework identified and discussed elsewhere [18]. One key limitation is the challenge of query composition, specifically construction of SPARQL queries using the SHARE client. While recent work has significantly eased the burden of creating SPARQL queries for ontologies that use alphanumeric identifiers by using the more human friendly labels during query formulation [32], more work is necessary to abstract this powerful, but technical interface so that it becomes more user-friendly to life scientists. In this respect, RDF graph browsing, possibly faceted, may offer a friendlier alternative to SPARQL query composition by allowing the user to dynamically migrate from one entity to another via incoming or outgoing properties. SADI is compatible with this approach because one can view the entirety of RDF data that can be generated by the collection of SADI services within a given repository, as one virtual RDF graph [14]. Practically, when a user visits a node, in addition to the links to catalogued data, the available properties that can be attached to the node by the services in the repository are also presented. If the user tries to follow such a link, calls are made to the appropriate services, and the generated data is added to the currently existing part of the RDF graph. Although this approach is not conducive to rapid automated classification of large amounts of lipids, it is efficient in informing the creation of integrated queries and the identification of the limitations and capabilities of the services present in a given SADI repository, in a user-friendly fashion. This approach has already been implemented as a plug-in to the Sentient Knowledge Explorer, a commercial data exploration and visualization system from IO Informatics [33].

Another viable possibility is manual or computer-aided composition of SADI services into workflows: an activity that can be characterized as programming without coding. This can be beneficial in fine-tuning service composition. A plug-in for the Taverna workflow management system that supports such use of SADI services is available [34]. With this plugin, the task of service composition is greatly facilitated: mapping of available starting data to service inputs in Taverna is semi-automated, and the system also suggests services that can apply to a piece of data returned by a service present in a given workflow, consequently the user only needs to look through short lists of applicable services. This allows the user to create complex queries and workflows that can potentially draw on services other than those already provided within the SADI framework. In short, the availability of such query tools increases the practical value of SADI as an integration medium for lipidomics resources, and bioinformatics resources in general.

The second aspect of the SADI framework that limits its rapid adoption is the challenge of creating semantic descriptions of services by SADI service providers [18]. This includes finding relevant ontological primitives in existing ontologies and making the chosen form of semantic description (service IO) compatible with the way related services are described, to ensure that new services can be seamlessly integrated with existing ones. In the current study this problem has been much less significant than it is for cases where semantic descriptions must facilitate the integration of services generated as a result of multiple disparate efforts. We attribute this to the consistent use of SIO predicates in all service descriptions generated as a result of this study. Being an upper level ontology, SIO provides for many of our practical semantic modeling needs, such as expressing parthood on molecules, specifying attributes and identifiers of entities, citing publications and DB records, among others. More importantly, being currently *de facto *the central ontology of SADI services, SIO provides a common vocabulary for many services in the SADI service network, and thus greatly alleviates the problem of semantic integration. Consistent use of SIO also greatly simplifies query formulation, identified earlier as an ongoing challenge [18], since the user needs to know few property predicates and classes specific to the lipid classification task - other more general primitives used in our queries can be used in many different contexts and thus are easier to remember.

The third feature of the SADI framework that is worthy of discussion is that SPARQL querying over a network of distributed SADI services is essentially a special case of data federation and is likely to exhibit some performance problems common to other data federation implementations. For example, in relational database federation, indexing remote tables in order to facilitate rapid relational algebra joins is a significant challenge. Even in cases where the resulting join of two tables may be small, computing the join may require sending a lot of data over the network. The SADI approach to service integration is not immune to this problem: there are services that may potentially generate large outputs and only fractions of these data may have to be sent as input to other services, which is a close analog of relational joins. Although this may become a significant potential limitation to the SADI approach in some cases, as our first and last use cases suggest, the majority of queries of relevance to lipid classification are computed rapidly because they only call services that generate small output. Even the query retrieving all proteins related to a lipid is computationally inexpensive since the SADI service producing the long list of proteins is called last and the size of its output is not multiplied by the computational cost of any downstream service calls.

Finally, there are several limitations to lipid classification that are specific to the pipeline presented here. Of particular note is the tight coupling of the functional group annotation service to the lipid ontology. Whenever novel patterns are identified as essential in formulation a lipid class definition, the functional group annotation service would have to be updated. For example, if a certain substructure is defined in the lipid ontology and is used in formulating the definition of a given class, the absence of the capacity of the functional group annotator service to detect this substructure in the input molecule will translate into classification inaccuracies with respect to the newly defined lipid class. Fortunately, it is not necessary to continually refine the existing annotator service, as new functional group annotator services with the same semantic definition of their functionality as the existing service could be generated and automatically integrated into the classification pipeline with SADI. This is afforded by the controllable capacity of SADI to call multiple services that are capable of producing similar annotations on a given entity, followed by a seamless integration of such annotations from disparate services. This allows third parties to extend our lipid ontologies, introduce novel annotations and functional groups that may be essential in lipid class definition, and still be capable of classifying lipids in the fashion demonstrated here, provided they also create supporting SADI services to expand the range of annotations necessary.

### Improving Lipid Ontologies and Databases

Although we have made resource integration a primary focus of this work, the novel chemical classification functionality and the opportunity to start evaluating established manually curated knowledge in public databases are also significant developments in this work. For the first time, and with minimal effort, we have been able to produce consistent, coherent, and logically rigorous definitions of chemical classes, which can transform the way chemical classification is carried out within the major chemical knowledge repositories. We illustrate that a shift in focus from the manual classification of individual molecules to the logical definition of the nature of chemical classes is not only feasible but that existing chemical ontologies and hierarchies can be dramatically expanded, improved, and potentially reused with relative ease. In addition our initial tests on a limited set of chemical entities are highly promising with respect to demonstrating the feasibility of this approach and with further expansion of our ontology, with formally defined classes, we shall be able to maintain and improve the accuracy of classification we have already achieved.

Moreover, we have also been able to identify cases where our simple structure-based approach was insufficient in producing an accurate classification. This is not an insurmountable problem, and explicit requirements for the integration of additional information can be easily addressed by adding to the class definition in OWL and supporting services or knowledge bases to ensure a correct class inference is made. In fact, the ability to identify cases where classification cannot be achieved by chemical structure characterization alone is a positive feature of our approach which drives the specification of a rigorous, unambiguous, and error-free chemical classification. As the lipid ontology matures and more data and web services are available to complement the solely structure-based definitions of lipid classes introduced here, we anticipate that the accuracy of the classification will improve.

We have also seen that our classification framework helps identify the exact compounds that have been misclassified, letting the chemical curator find and correct either the definitions used for classification or the asserted class membership. In the process of creating the Lipid Eicosanoid Ontology used for classification here, we have experienced first-hand the instant feedback that the classification results provide for the refinement of chemical class definitions, correcting and improving our classification results to the level of accuracy reached here. We surmise a yet higher accuracy is within reach, however the current performance is sufficient for the purposes of demonstrating the methodology with the prototype classification and annotation pipeline presented here.

### SADI in Perspective: Evolution of Web Service-Based Resource Integration

The use of Web services for integration of distributed resources is a relatively popular research topic that can be discussed at length, so we shall only refer the reader to some related work that is either closely related to SADI or is based on well-known Web service technologies, such as SOAP and REST. The approach most closely related to SADI is that of BioMOBY [35] - a direct predecessor of SADI. Many core features of SADI were already present in BioMOBY. For example, in BioMOBY, messages exchanged between clients and services carry their own semantics and the decentralised use of ontologies is enabled. One of the advantages of SADI over BioMOBY is the use of RDF for the data model and format for service IO, whereas BioMOBY defines its own XML-based format. As a consequence, SADI service providers and consumers can choose from a variety of RDF API and tools to use in combination with the SADI software.

The key advantage of SADI is that the relation between service inputs and outputs is explicitly ontologically defined, in contrast to BioMOBY which *merely allows to semantically categorise service operations and IO *by specifying the ontological type of the service operation and the ontological types of the data the service expects as inputs and produces as output. Such categorisation (i) allows querying of service registries for services that provide functionality of specified types and acting on data of specified types, and (ii) helps identify services that can be combined, based on their IO types. BioMoby's categorization of service operation is however insufficient to support absolutely automatic service discovery and invocation for query execution. Although all our use cases can be solved by writing BioMOBY services and combining them in pipelines, the creation of the pipelines would require manual work by a bionformatician who would first identify the types of operations based on the use case requirements, and select the appropriate services, based on their textual description. For a more detailed comparison of SADI with BioMOBY and some other related approaches, we refer the reader to [14].

It is also appropriate to mention the two best known attempts to extend traditional Web service technologies with semantic descriptions. SAWSDL [36] extends the Web Service Description Language (WSDL), typically used to provide machine-processable descriptions of SOAP services - with references to classes from external ontologies. Likewise, SA-REST [37] specifies a way to assign such annotations to RESTful services. These frameworks are gaining popularity in the bioinformatics community because they are supported by the Biocatalogue [38] registry of Life Science web services. Both approaches implement semantic categorisation in the same fashion as BioMOBY, so they also suffer from the corresponding limitations described earlier. Moreover, in comparison with BioMOBY, they have an additional problem - the necessity for data mediation. Since neither SAWSDL nor SA-REST standardise the IO data formats, they require service providers to write special XSLT rules for converting native service IO data to and from RDF to enable composition of services with incompatible IO data types.

Finally, we would like to mention the possibility of wrapping existing SOAP and RESTful services as SADI services. When service migration to SADI is impossible or impractical, one can sometimes write a proxy SADI service that converts the input RDF into a native input for the SOAP or RESTful service being wrapped, and converts the output of the service back to RDF. This kind of conversion may be easier for services with SAWSDL or SA-REST descriptions because they already have semantic types assigned to input and output and, possibly, RDF conversion rules in XSLT, albeit the relation between input and output still needs to be specified.

### Future Developments

A limitation of our classification scheme is the inability to handle molecular classes that are defined in part by some feature of chemical functionality (e.g. toxicity or mutagenicity). We have only demonstrated an efficient framework to handle structure-based classification here, and would like to handle functional classification in a separate work. However, functional classification is still possible with our approach; it merely requires a different set of molecular annotations in order to adequately support classification. For instance, the class of oestrogen receptor agonists may involve some consistent chemical substructures or properties, but the underlying feature of the class is the presence of an EC_50 _value annotation on a given molecule and the location of this value within a given numerical range. Our framework allows for formulation of classes with respect to a broad range of annotations, so functional classification is only a matter of adequately formulating the definition of a given functional class, and seeking the appropriate underlying molecular descriptors. We understand that some molecules may be inconsistently classified by human experts for historical reasons (e.g. a compound erroneously believed to have therapeutic effects), or due to the broad definition of chemical classes that may invite ambiguities or inconsistencies. However, we do not foresee this as an insurmountable challenge; it merely awaits careful consideration and contribution to the specification of formal definitions by the relevant communities of researchers. Given the flexibility, the ability to improve accuracy, and the extensibility of class definitions in OWL, as well as the ease of integration of these logical definitions into chemical classification with SADI-enabled service interoperability, we believe that our approach can be successfully expanded to include all of LIPID MAPS and many other primarily structure-based classification schemes. In the future, we intend to expand our framework to adequately treat the entirety of the LIPID MAPS hierarchy and to enable automated classification for the entire lipidomics domain, further expanding and contributing to the ecosystem of novel *in silico *applications in this domain [39].

## Conclusions

Chemical classification and facile integration of disparate computational and information resources is of immense value to the interdisciplinary and highly interlinked future of life sciences research, especially -omics and systems level investigations. The framework presented here provides a potential means for the integration of computational and informational resources and leaves room for further expansion and improvement of chemical knowledgebases and ontologies. Given our results, we strongly believe that the principles and approaches we demonstrate here can form the foundation for future knowledge integration and integrative analysis in life sciences.

## Methods

### Semanticscience Integrated Ontology

The Semanticscience Integrated Ontology (SIO) provides basic classes and relations for semantic annotation of data and services. This OWL2 ontology consists of 800+ classes across three major categories (physical entity, processual entity and informational entity) from which domain specific sub-types can be further specified. The ontology contains 1 'has value' datatype relation to store literals and 129 object relations pertaining to membership and parthood, spatial positioning, temporal ordering, representation and aboutness, attributes, qualities, participation and agency. The ontology, adopted as the basis for semantic interoperability across SADI-compliant services, provides a basic vocabulary for constructing sophisticated class expressions to describe SADI service inputs and outputs, which can checked for consistency through automated reasoning. Specifically, our work made use of object relations involving parthood (SIO_000053) and informational attributes (SIO_000008) and use the 'has value' datatype relation (SIO_000300) to specify values.

### Functional Group Annotation of Lipids

The functional group annotation service [20] operates upon molecules possessing a defined chemical graph structure that is specified as a SMILES string. Substructures present in the LiPrO ontology and the Lipid Eicosanoid Ontology (LEO) and relevant to chemical classification were defined using the SMARTS chemical pattern specification [40]. This specification allows the definition of generic chemical graph structure. SMILES-encoded lipid chemical structures were parsed using the Chemistry Development Kit [41] and subsequently queried for matches to one of the 155 SMARTS patterns. The matches were then filtered for only unique and non-overlapping substructures. Since some substructures (e.g. the carbon atom) are present in overwhelming numbers within the lipids, and since the cardinality on such functional groups is not required to define any of the classes used herein, only one instance of such substructures was reported. As output, the service reports substructures as instances of corresponding functional groups and as part of the queried chemical entity.

### Reasoning over Lipid data for Classification using Lipid Ontology

The OWL API [42] and Pellet OWL 2 Reasoner for Java [43] were used to implement ontology-based lipid classification service. The input for the service includes the lipid ontology and a description of the lipid in the form of its functional groups. The output is a list of lipid class names identified using the following procedure. First, the input description is converted into an OWL anonymous class expression formed as an intersection of existential and cardinality restrictions on the parthood relations, reflecting the existence and/or the precise number of functional groups present in the molecule. For example, if the input specifies exactly three alkenyl groups and a carboxylic acid group, the corresponding members of the intersection will be ('has proper part' exactly 3 Alkenyl_Group) and ('has proper part' some Carboxylic_Acid) respectively. Second, in a loop for each named lipid class from the ontology, the reasoner is called to check if the named class subsumes the anonymous class representing the input. Named classes that are recognized as superclasses of the anonymous input class are saved in the output set which is later pruned by removing classes that are superclasses of other classes in the set, so that only the most specific predicted classes are reported.

### Lipid Eicosanoid Ontology

The Lipid Eicosanoid Ontology (LEO [44]) is an OWL ontology that is derived from the earlier reported LiPrO general lipid ontology to specifically address the eicosanoid hierarchy as defined by LIPID MAPS. Since the LiPrO ontology was initially geared towards a broader class of lipids and was not specifically designed with eicosanoids in mind, it was missing a number of chemical pattern definitions and axioms that were indispensable in classification of eicosanoids, as well as a number of eicosanoid classes. In the creation of LEO, formal logical definitions for each eicosanoid class in LIPID MAPS were generated manually upon careful examination of the corresponding parts of the asserted LIPID MAPS hierarchy to identify the consensus structural patterns, as well as any restrictions on their occurrence and configuration. To do this, we generated a set of chemical substructures of relevance to eicosanoids and screened this set to identify the substructures present in all representatives of a given class, using Chemistry Development Kit. In addition to this, to inform cardinality restrictions, we derived metrics on the minimal count of unique non-overlapping occurrences of these consensus substructures in members of each lipid class. All the chemical structural patterns previously defined in LiPrO have been preserved in LEO, while the complete lipid class hierarchy of LiPrO has been removed in favour of a refined eicosanoid lipid class hierarchy. To accommodate the logical definitions of eicosanoid classes that were previously absent in LiPrO, 16 new structural patterns were defined in LEO. The methodology of class definition construction in LEO has also been preserved from LiPrO. Thus, class expressions in LEO are defined in terms of a combination of the substructures that are both necessary and sufficient for classification. For example, a fatty acid is defined as a molecule that contains an alkyl chain or a primary acyl chain, a carboxylic acid derivative, and a chain of three carbon atoms that are not members of a ring. The construction of such class definitions somewhat resembles the construction of chemical class fingerprints in classical cheminformatics, where bits in a fingerprint are set to either true or false, depending on absence or presence of a given chemical feature. Unlike the classical chemical class fingerprints, the use of OWL allows a much enhanced expressivity of chemical class definitions and expands the diversity of concepts and features that one may draw upon in order to define lipid classes. For instance, the use of OWL has enabled us to specify the requirement of the presence of a certain minimal or exact number of specific substructures via qualified cardinality restrictions in LEO. This is important in defining membership in the epoxyeicosatrienoic acid class, among others, which requires a lipid to possess exactly three alkene substructures, with variations in their distribution that cannot be reflected in classical chemical fingerprints. Finally, in order to ensure semantic interoperability of LEO terms, we have employed relations from the Semanticscience Integrated Ontology, which is an upper level ontology used within the SADI framework, in place of the relations earlier used in LiPrO.

### Evaluation of Classification

The accuracy of chemical classification for 150 eicosanoids from the LIPID MAPS database (October 19, 2010) was determined by comparing the results of automated reasoning with the LEO ontology against the manually curated LIPID MAPS classification. This evaluation set was selected randomly out of a much bigger set of eicosanoids. The rest of the eicosanoid set was used for the purposes of informing and refining the formal definitions of lipid classes in LEO, as described. In order to carry out the evaluation, we created a standalone classifier program that uses the OWL API (version 3.1.0) to parse the LEO ontology populated with molecular data and call the Pellet Reasoner (version 2.2.2) to infer the class membership of instances of molecules. To ensure reasoning over qualified cardinality restrictions, additional statements were added to specify that the molecule only contained the functional groups identified by the annotator service and that all of the instantiated individuals were distinct. The effectiveness of classification was evaluated by tabulating the exact matches, partial matches and mismatches between the inferred and manually assigned class membership. Partial matches included cases where our framework was incapable of producing an exact match, but still produced a classification that was not inconsistent with manually curated classification. For example, assignment of a lipid to the parent class of its manually curated class constituted a partial match. Mismatches comprised of LIPID MAPS-asserted members of one terminal class (class without further child classes) being assigned to an incorrect terminal class.

### Text Annotation Service

The text mining solution [9] is based on open source General Architecture for Text Engineering (GATE) framework [45]. GATE comes with many plug-ins and processing resources by default, where one of them is the ANNIE (a Nearly-New Information Extraction System) component [46]. ANNIE can be used for common natural language processing tasks such as tokenization, sentence splitting and processing of gazetteer lists. A GATE pipeline was created that includes an ANNIE gazetteer as well as an ANNIE tokenizer and an ANNIE sentence splitter for advanced text processing. Gazetteer lists [9] were used to identify mentions of lipids in a text by mapping synonyms of lipid names to LiPrO classes. The pipeline accepts ASCII text and lipid gazetteer lists as input, while the output consists of a list of identified lipids specified by their canonical names and LiPrO classes. The pipeline can be used as a Java API or as a SADI service that takes a document URL and annotates it with the identified lipid classes in the output.

## Availability and requirements

A detailed explanation for accessing the web services described herein, along with sample input files, is available [24], and SPARQL queries of the framework constructed here, along with the entirety of the SADI web service collection, can be posted at the web interface referenced in [24], which is accessible without restrictions through a web browser. The collection of the services we have created here can also be freely accessed by direct HTTP POST and (with the sample input files we provide) GET operations to the service addresses [20,22,25,26,28-30].

## Competing interests

The authors declare that they have no competing interests.

## Authors' contributions

LLC and MD created the LEO ontology used in this study, extracted and expanded from the original LiPrO ontology created by HSL and CB. LLC developed the functional group annotation software, including the corresponding SADI service. AK and LLC wrote different versions of the classifier code. LLC carried out the validation of classification. AK created the lipid text mining pipeline. AR created the SADI services except the functional group annotation service, and conducted the experiments with SHARE. LLC, CB and AK conducted the preliminary evaluation of lipid classification. CB and MD coordinated the work. All authors except HSL contributed to the final manuscript. All authors read and approved the final manuscript.
